# College Organizational Innovation Performance-Oriented Internal Mechanism Analysis Using Lightweight Deep Learning under Health Psychology

**DOI:** 10.1155/2022/6207027

**Published:** 2022-06-20

**Authors:** Mengqiong Liu

**Affiliations:** School of Economics and Management, Ningxia University, Yinchuan 750021, China

## Abstract

The purpose is to improve employees' initiative and innovation performance and further improve the overall organizational efficiency of colleges. From the perspective of health psychology, this work analyzes the internal mechanism between leadership empowerment behavior and employee innovation performance at China Agricultural University. By introducing two intermediate variables: task-based psychological capital (PsyCap) and innovative PsyCap, this work puts forward a lightweight deep learning (DL) model. It constructs the college organizational innovation performance (OIP)-oriented internal evaluation system from four dimensions. They are personal development support, power appointment, participation in decision-making, and work guidance. Then, the proposed lightweight DL model reveals the internal relationship between employees' innovation performance and innovation factors using the questionnaire survey method. Overall, 360 questionnaires are distributed. The results show that the average values of the four dimensions (*S*, *P*, *D*, and *G*) of leadership empowerment are greater than 3, which are 4.3144, 4.3493, 4.4253, and 4.5286, respectively. S, P, *D*, and *G* represent empowerment, decision-making, communication, and innovation, respectively. The results show a high level of innovation performance in all dimensions. The finding proves that the influencing factors of employee innovation performance mainly include personal development support, empowerment, participation in decision-making, and work guidance. The effects of different dimensions vary significantly. Finally, the lightweight DL model can improve the analysis accuracy of the college OIP-oriented internal evaluation system. Therefore, college leaders should use the DL model and empowerment behavior to improve employees' psychological quality, innovation enthusiasm, and work efficiency, ultimately benefiting employees.

## 1. Introduction

In the context of deep learning (DL), the flat organizational structure (FOS) has gradually replaced the traditional organizational structure. The traditional organizational structure is hierarchical based on centralized decision-making and top-down management and control concepts. The DL-based performance evaluation system (PES) can enhance the new organizational vitality in the organizational innovation of colleges and universities (CAUs). The leadership is responsible for determining and organizing employees' work, making important decisions, and providing incentives. The staff is only responsible for completing the tasks assigned by the supervisor [1, 2]. The FOS objects to the classification and strict control management of the traditional organizational structure. It pays attention to the independent initiative of employees, which helps to simplify the organization, reduce costs, and improve efficiency. Therefore, many organizations adopt the behavior of empowerment, which requires the leaders in the organization to give corresponding power down to promote employees' self-motivation [3, 4]. Practice proves that the leader's empowerment behavior can promote the enthusiasm and innovation ability of employees in the organization. At the same time, organizational leaders' empowerment behavior also affects employees' work conscientiousness, work enthusiasm, and workability based on health psychology [5]. For example, Sun et al. [6] studied the performance improvement path of project management from the perspective of configuration and used the qualitative comparative analysis to explore the improvement path of project management performance. They found that there were four combination paths that could improve project management performance, and the research could promote the development of project management performance from simplification to comprehensiveness.

On this basis, combined with the actual situation of colleges and universities, the internal mechanism of empowerment behavior and employee innovation performance in colleges and universities is explored based on psychology. Wen et al. [7] revealed that the current situation of the leadership of college managers was analyzed through a questionnaire survey. The role change and the influence of college leadership empowerment behavior on employee innovation performance are discussed. The internal mechanism of psychological capital (PsyCap) is studied, and its intermediary role is discussed. This work provides a strong reference for some organizations in the future. The transformation of the organizational model and the upgrading of organizational management have a guiding role. At the same time, they also guide the leaders of the organization on how to improve the enthusiasm and innovation of all employees in the organization through psychology, achieving better and faster development of the organization. Additionally, the research results of relevant scholars show that the lightweight DL model has great application potential. Agarwal et al. [8] studied the lightweight DL model of human behavior recognition (HBR) on edge devices. Through HBR, the research could improve the efficiency of resource deployment and resource utilization on edge devices. Karakanis et al. [9] studied coronavirus disease 2019 (COVID-19) detection DL model by developing the generative adversarial network (GAN). The robustness of adversarial input was demonstrated in binary and multiple class cases. Chiu et al. [10] predicted the real estate price based on the lightweight DL model. A novel spatiotemporal influence diagram was designed, and the computational cost of this model was much lower than that of the traditional model, which was suitable for practical application. The lightweight DL model could use the computer to analyze the system data and improve the innovation management level of organizational innovation performance in CAUs. The deficiency was that this literature model consumed high computation, difficult to be widely used.

Based on the literature review, this work innovatively conducts an in-depth study on empowered leadership in the context of the scarcity of relevant domestic research. The previous research on organizational leadership behaviors mainly focused on transformational leadership (TL) and power TL. Their content, structure, cause, and effect are self-explanatory. However, there is little research on EL in China. With the development of FOS and the improvement of employees' knowledge, people have higher expectations for this EL. The research motivation is to provide strong support for the research related to EL. The main contribution is to enrich relevant research content and provide a reference for researching organizational empowerment behaviors.

## 2. Research Hypothesis and Questionnaire Measurement

### 2.1. The Hypotheses Proposed

PsyCap plays an intermediary role between leadership empowerment behavior and employee innovation performance. Combined with the relevant data of the lightweight DL model [11], based on many variables (personal development support S, power appointment P, participation in decision-making *D*, work guidance G, employee innovation action IA, employee innovation effect IE), the hypotheses are put forward and listed in Table 1.

The hypotheses related to the task PsyCap (*T*) are as follows:

- H1-*a*: between *S* and *IA*
- H1-*b*: between *P* and *IA*
- H1-*c*: between *D* and *IA*
- H1-*d*: between *G* and *IA*
- H1-*e*: between *S* and *IE*
- H1-*f*: between *P* and IE
- H1-*g*: between *D* and IE
- H1-*h*: between *G* and *IE*

The hypotheses of innovative PsyCap (I) are as follows:

- H2-*a*: between *S* and *IA*
- H2-*b*: between *P* and *IA*
- H2-*c*: between *D* and *IA*
- H2-*d*: between *G* and *IA*
- H2-*e*: between *S* and *IE*
- H2-*f*: between *P* and *I*
- H2-*g*: between *D* and *IE*
- H2-*h*: between *G* and *IE*

### 2.2. Variable Measurement

This work uses the method of an online questionnaire to collect data and uses empirical research to verify the relationship between leadership empowerment behavior, employees' psychological quality, and innovation performance. The questionnaire consists of four parts: subjects' basic information, leadership empowerment behavior measurement, PsyCap measurement, and employee innovation performance measurement. Domestic scholars have modified the scale of three variables based on the original scale to obtain good reliability and validity, which align with this work [12, 13]. The scoring rules of the research scale are listed in Table 2.

Then, the detailed information of the questionnaire should include the following aspects:

1. Basic information on subjects includes age, gender, educational background, and department.
2. Measurement of leadership empowerment behavior. This part is based on evaluating senior leadership behavior in the leadership empowerment scale prepared by Feng and Liu [14]. According to the actual research background and perspective, this work selects four dimensions: personal development support, empowerment, participation in decision-making, and work guidance.
3. Measurement of psychological capacity. This part measures employees' mental health, which mainly draws lessons from Ali et al., mental health scale [15].

### 2.3. Sample Selection and Data Collection

The survey subjects are students who have been involved in innovation and entrepreneurship from four different universities in Shaanxi Province to ensure the applicability of the survey data. The questionnaire was distributed to the subjects on the Questionnaire Star Platform, and the finished test was collected. After two months, 360 questionnaires were distributed, and 340 were recovered. SPSS 22.0 statistical analysis software is used to analyze the effective data, including descriptive statistical analysis of variables, questionnaire reliability analysis, correlation analysis, and hypothesis test.

## 3. Empirical Research Analysis

### 3.1. Descriptive Statistical Analysis of Samples

#### 3.1.1. Descriptive Statistical Analysis of Leadership Empowerment Behavior

SPSS 22.0 software is used for descriptive statistical analysis of the dimensions of leader empowerment. The results show that the average value of the four dimensions of leadership empowerment is greater than the median 3. These four dimensions are *S*, *P*, *D*, and *G*, representing empowerment, decision-making, communication, and innovation, respectively. The average values of the four dimensions are 4.3144, 4.3493, 4.4253, and 4.5286, respectively. The results show that the superior leaders of the research object are outstanding in *S*, *P*, *D*, *G*, and other aspects, and there are obvious authorization and empowerment behavior in work. The details are as follows.

**Figure 1:**
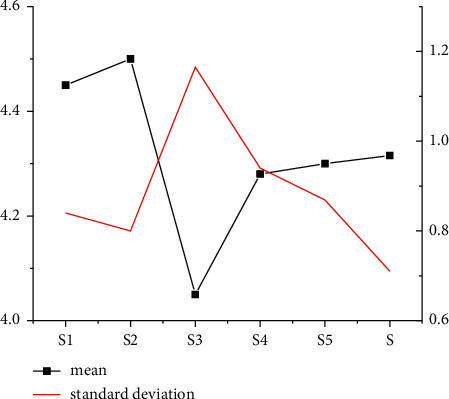
Descriptive statistical analysis of *S* dimension.

**Figure 2:**
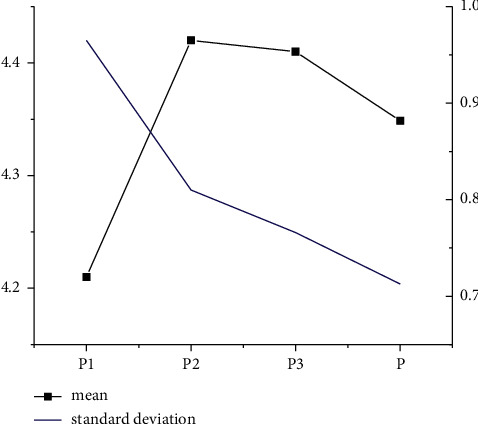
Descriptive statistical analysis of *P* dimension.

As Figures 1 and 2 indicate, under the dimensions of S and P and the descriptive statistical analysis, standard deviations of *S*1, *S*3, and *S*4 are higher than the average value, while the standard deviations of *S*2 and *S*5 are less than the average value. The average analysis factor of the S dimension is 1.0. In the P dimension, the standard deviations of *P*2 and *P*3 are below the average statistical analysis results, and the standard deviation of P1 is higher than the average statistical analysis result.

**Figure 3:**
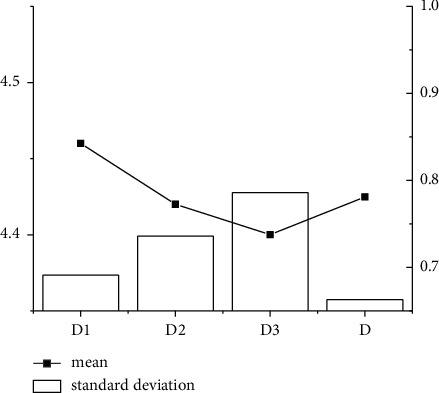
Descriptive statistical analysis of D dimension.

**Figure 4:**
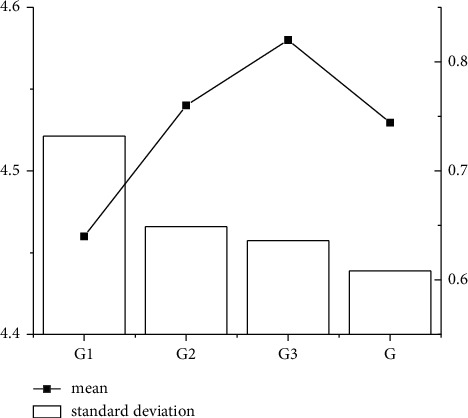
Descriptive statistical analysis of G dimension.

From the descriptive statistical analysis results in Figures 3 and 4, the average descriptive analysis factor is higher than the standard deviation in dimension *D*. In contrast, in dimension *G*, only the descriptive factor of *G*1 is higher than the standard deviation. The other descriptive analysis factors are lower than the average deviation.

#### 3.1.2. Descriptive Statistical Analysis of Psychological Capital

According to the descriptive statistical analysis of PsyCap, the average value of *T* is 4.5642, and the average value of *I* is 4.5642, indicating that the subjects in this work have high PsyCap in terms of task completion and innovation.

**Figure 5:**
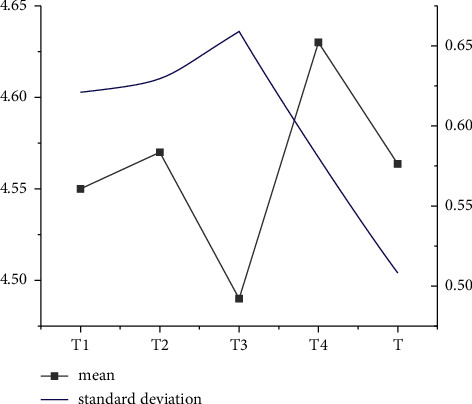
Descriptive statistical analysis of *T* dimension.

**Figure 6:**
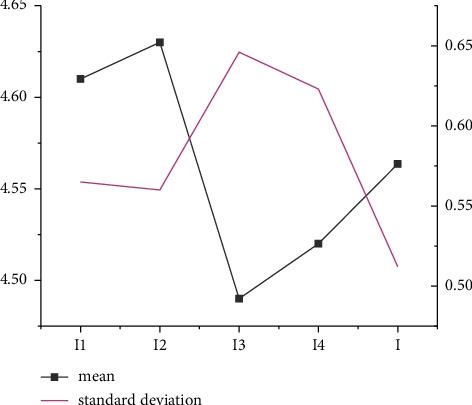
Descriptive statistical analysis of *I* dimension.

Figures 5 and 6 show that the average result of descriptive statistics in dimensions *T* and *I* is about 0.6 and 0.55, respectively. In addition, the standard deviation of descriptive statistics in *T* and *I* dimensions is about 4.6 and 4.55, respectively.

#### 3.1.3. Descriptive Statistical Analysis of Employee Innovation Performance

**Figure 7:**
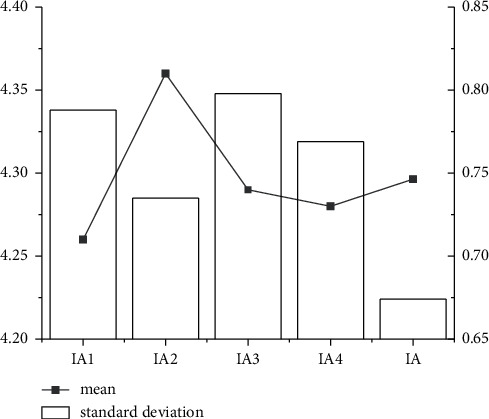
Descriptive statistical analysis of IA dimension.

**Figure 8:**
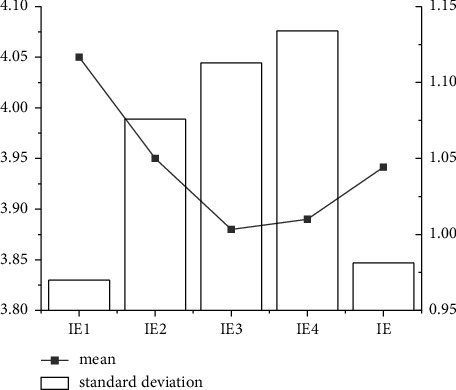
Descriptive statistical analysis of IE dimension.

Through the descriptive statistical analysis of the two dimensions of employee innovation performance, it is found that the subjects have a high level of innovation performance in both IA and IE. At the same time, the average IA is 4.2957, greater than the average IE. This may indicate that the subjects have high levels of innovative action at work, but innovative results are difficult to obtain. The details are illustrated in Figures 7 and 8.

As shown in Figures 7 and 8, the average result of descriptive statistics in IA and IE dimensions is about 0.75 and 1.05, respectively. In addition, the standard deviation of descriptive statistics in the IA and I dimension is 4.3 and 3.95, respectively. Combining the descriptive statistical results of different dimensions reveals that the evaluation of the IA dimension is more accurate than that of the IE dimension.

### 3.2. Questionnaire Reliability Analysis

Reliability refers to the consistency of repeated measurement results of the same subject using the same test tool under the same conditions. The higher the consistency is, the higher the reliability is, which reflects the reliability and stability of the test tool. Reliability analysis is the basis of validity analysis, so the reliability analysis of empirical questionnaire research is very important. In this work, Cronbach's *α* coefficient method is commonly used in the reliability analysis of the questionnaire [16–18].

SPSS 22.0 software is used to analyze the internal consistency of three variables and their dimensions of leadership empowerment behavior, PsyCap, and employee innovation performance, and the coefficients of each dimension and the whole scale are obtained, as shown in Table 3. The table shows that Cronbach's *α* coefficient of leadership empowerment behavior scale is 0.958, Cronbach's *α* coefficient of PsyCap scale is 0.937, and Cronbach's *α* coefficient of employee innovation performance scale is 0.962, which are all greater than 0.9. Cronbach's *α* coefficient of each dimension of the variable is the lowest 0.856 and the highest 0.948, which shows that the questionnaire has high reliability, strong internal consistency, and strong reliability [19, 20].

### 3.3. Correlation Analysis

Correlation analysis is a statistical analysis method used to measure the correlation between two or more variables in the same class [21, 22]. In this work, the Pearson correlation coefficient method and SPSS 22.0 software are used to analyze the correlation between variables. Pearson correlation coefficient is between (−1, 1), and the correlation between the two variables is proved. The correlation coefficient between variables is shown in Table 4.

Table 4 shows that the correlation coefficient between variables is between 0 and 1, which is significant at the 0.01 level. Among them, the correlation coefficients between S, P, *D*, G, and T are 0.471, 0.498, 0.488, and 0.488, respectively, indicating that the task PsyCap of employees is positively affected by the empowerment of leadership. The more the work guidance behavior there is, the higher the level of task PsyCap is. Similarly, these four dimensions are positively correlated with *I*, IA, and IE, and *T* and *I* are positively correlated with IA and IE. The correlation between variables in this work is consistent with the previous research hypothesis.

### 3.4. Hypothesis Testing and Results Summary

The mediating effect of PsyCap is verified. The operation is as follows: first, the data are processed, and personal support development (CS), power appointment (CP), participation in decision-making (CD), work guidance (CG), task-based PsyCap (CT), innovative PsyCap (CI), innovative action (CIA), and innovative effect (CIE) are named. Then, PROCESS is loaded into SPSS 22.0, the data are imported, and the mediation analysis is conducted.

#### 3.4.1. Test on the Mediating Effect of PsyCap on Personal Development and Innovation Performance

When it exists between CS and CI, the PsyCap is tested, and the results are shown in Table 5 and Table 6.

Table 5 shows that CS has a significant impact on CI in the regression results of independent variables on mediating variables. The effects of CS on CIA and CI on CIA are significant in the regression results of independent variables and mediating variables on dependent variables. Subsequently, the mediating effect of CI is tested, the BootLLCI value of CI is 0.2451, the BootULCI value of CI is 0.4022, and the value range does not contain 0. The study shows that CI plays an intermediary role between CS and CIA under H2-a. And the influence of independent variables and dependent variables is observed after the intermediary variables are limited [23]. When the test values are between 0.0877 and 0.2972 without 0, CI plays a partial mediating role between CS and CIA.

The mediating effect of CI on the relationship between CS and CIE is analyzed by the same step. It is found that the mediating effect of CI is between (0.2033, 0.2983) under the influence of independent variables and mediating variables, and the hypothesis that H2-e played a mediating role is proved. After the role of CI is controlled, CS has a significant direct impact on CIE, and CI is not the only intermediary.

#### 3.4.2. Test on the Mediating Effect of PsyCap on Power Appointment and Innovation Performance

Table 7 shows that CP is the independent variable, CIA is the dependent variable, CT is the intermediary variable 1, CI is the intermediary variable 2, and CP has a significant effect on CT and CI. At the same time, CP, CT, and CI have a significant impact on innovation activities, and the intermediary path can be verified. The verification of CT is between 0.0833 and 0.1799, and the verification of CI is between 0.2153 and 0.2782, which indicates that they have a great influence on the relationship between CP and CIA. This proves H1-b and H2-b. After the mediation is controlled, CP has a significant effect on CIA, so the mediation between the two is not unique [24, 25].

Table 8 shows that these two kinds of PsyCap are affected by CP, and the influence of intermediary variables on independent variables and dependent variables is different from the regression results. The mediating effect of CT ranges from -0.0502 to 0.2782, including 0, indicating that the task-based effect has no obvious PsyCap on innovation, and power constraints and innovative PsyCap have a significant impact on innovation effect. The test results of the intermediate path show that the BootLLCI value of CT is −0.0318, and the BootULCI value is 0.0862. The test range includes 0, indicating that Hypothesis H1-f is not correct. The BootLLCI value of CI is 0.1882, and the BootULCI value is 0.2671. The test range does not include 0, indicating that CI between CP and CIE has a mediating effect. The H2-f hypothesis is proved. After the mediating effect is controlled, the dependent variable is directly affected by the independent variable, indicating that CI is not the only intermediary [26, 27].

#### 3.4.3. The Test on the Mediating Effect of PsyCap on Participation in Decision-Making and Innovation Performance

The mediating effect of task-based PsyCap and innovative PsyCap on participation in decision-making and innovation performance is shown in Tables 9 and 10.

Table 9 shows that CD has a significant impact on CT and CI. The regression results of independent variables and mediating variables on dependent variables show that CD, CT, and CI have significant impacts on CIA. In the mediating path test, the mediating test value of CT is between (0.0832, 0.1704), and the mediating test value of CI is between (0.1709, 0.2733). If H1-c and H2-c are effective, CT and CI play a mediating role between CD and CIA [28]. After the mediating variables are controlled, the direct impact of independent variables on the dependent variables is observed. The LLCI value is 0.0122, and the ULCI value is 0.2361, indicating that CIA is significantly affected by CD, so CT and CI are not the only intermediaries between the two.

Table 10 shows that CD has a significant effect on CT, CI, and CIE, in the mediating effect test of PsyCap on CD and CIE. But the test range of the mediating effect of CT on CIE included 0 and does not pass the effect test of the mediating path, so H1-g is not correct. The results show that CI usually has a very significant effect on CIE. In the intermediary path, the BootLLCI value is 0.866, and the BootULCI value is 0.2942, indicating that CI is the bridge between CIE and CD. If H2-g is correct, the dependent variable will change significantly with the change of independent variables after the mediation is controlled, which also shows that CI usually has only a certain mediation effect [29–31].

#### 3.4.4. The Test on the Mediating Effect of PsyCap on Work Guidance and Innovation Performance

In the verification of the mediating effect of PsyCap on work guidance and innovation performance, there is a negative correlation between CG and CI, and the effect is not obvious, which does not match the test index of the mediating effect [32–34], so H2-d and H2-h are not correct. Therefore, only the mediating role of task-based PsyCap between work guidance and innovation performance is examined, and the results are shown in Tables 11 and 12.

Table 11 shows that the intermediary test value of CG on CT is between 0.3589 and 0.4731, excluding 0, which shows that the influence of CG on CT is significant. The regression results of independent variables and intermediary variables on dependent variables show that CIA is significantly affected by work guidance and CT [35, 36]. In the mediation verification, the BootLLCI value of CT is 0.1755, the BootULCI value is 0.4032, and the mediation test value is between (0.1755, 0.4032), which do not contain 0, indicating that CT has a significant intermediary role between CG and CIA, and only plays a certain intermediary role. This shows that H1-d is true.

Similarly, the test results of Table 12 show that CT has a significant mediating effect between CG and CIE, and H1-h is supposed to be correct. After the mediation effect of CT is controlled, CG has a significant direct impact on CIE, and the mediation between the two is not unique.

## 4. Conclusion

This work is conducted in the form of a questionnaire. The samples are selected and screened through the questionnaire survey and research hypothesis. The questionnaire's reliability and validity are tested by descriptive statistical analysis. Finally, it analyzes the intermediary role of task-based PsyCap in work guidance and innovation performance. The research results show that the three dimensions of empowerment and innovation positively impact the development of employees' decision-making. Therefore, leadership empowerment promotes innovative behavior and results by affecting employees' work attitudes and PsyCap. PsyCap is not the only intermediary between leadership empowerment and employees' innovation performance. The research provides a powerful path for organizational leaders to motivate employees' innovative performance. In organizational management, managers' empowerment behavior is of great value to improve the organization's innovation performance. A healthy and sustainable organization should strive to create a positive and comfortable environment and working atmosphere, enhance the enthusiasm and innovation of employees, and promote the development of the organization. Finally, some shortcomings need to be improved. The main deficiency is that the calculation cost of the model is too high, and the research results might not be generalizable. Therefore, the later research will collect more data, optimize the model, reduce resource consumption, and improve the operation efficiency of the system.

## Figures and Tables

**Table 1:** Variable hypothesis based on lightweight DL model.

	*H*1	*H*2
*a*	S ∼ IA	S ∼ IA
*b*	P ∼ IA	P ∼ IA
*c*	D ∼ IA	D ∼ IA
*d*	G ∼ IA	G ∼ IA
*e*	S ∼ IE	S ∼ IE
*f*	P ∼ IE	P ∼ IE
*g*	D ∼ IE	D ∼ IE
*h*	G ∼ IE	G ∼ IE

**Table 2:** Questionnaire item and scoring criteria.

Primary dimension	Secondary dimension	Scoring criteria
Attitude	Performance evaluation	0.30
	Empowerment behavior	0.13

Ability	Entrepreneurial experience	0.05
	Entrepreneurial scope	0.27

Knowledge	Policy understanding	0.05
	Innovation output capacity	0.20

**Table 3:** Cronbach's *α* coefficient of each variable.

Variables	Dimensions	Cronbach's *α* coefficient of each dimension	Cronbach's *α* coefficient of the scale
Leadership empowerment behavior	Personal development	0.902	0.958
	Power appointment	0.856	
	Participation in decision-making	0.928	
	Work guidance	0.925	

PsyCap	Task-based PsyCap	0.887	0.937
	Innovative PsyCap	0.915	

Innovation performance	Innovation action	0.933	0.962
	Innovation effect	0.948	

**Table 4:** The matrix of Pearson correlation coefficient.

	*S*	*P*	*D*	*G*	*T*	*I*	*IA*	*IE*
S	1	0.695 ^*∗*^ ^*∗*^	0.688 ^*∗*^ ^*∗*^	0.733 ^*∗*^ ^*∗*^	0.472 ^*∗*^ ^*∗*^	0.455 ^*∗*^ ^*∗*^	0.453 ^*∗*^ ^*∗*^	0.453 ^*∗*^ ^*∗*^
P	0.697 ^*∗*^ ^*∗*^	1	0.714 ^*∗*^ ^*∗*^	0.705 ^*∗*^ ^*∗*^	0.498 ^*∗*^ ^*∗*^	0.518 ^*∗*^ ^*∗*^	0.469 ^*∗*^ ^*∗*^	0.393 ^*∗*^ ^*∗*^
D	0.685 ^*∗*^ ^*∗*^	0.711 ^*∗*^ ^*∗*^	1	0.726 ^*∗*^ ^*∗*^	0.502 ^*∗*^ ^*∗*^	0.497 ^*∗*^ ^*∗*^	0.447 ^*∗*^ ^*∗*^	0.388 ^*∗*^ ^*∗*^
G	0.733 ^*∗*^ ^*∗*^	0.706 ^*∗*^ ^*∗*^	0.727 ^*∗*^ ^*∗*^	1	0.483 ^*∗*^ ^*∗*^	0.405 ^*∗*^ ^*∗*^	0.382 ^*∗*^ ^*∗*^	0.302 ^*∗*^ ^*∗*^
T	0.471 ^*∗*^ ^*∗*^	0.498 ^*∗*^ ^*∗*^	0.488 ^*∗*^ ^*∗*^	0.488 ^*∗*^ ^*∗*^	1	0.667 ^*∗*^ ^*∗*^	0.609 ^*∗*^ ^*∗*^	0.411 ^*∗*^ ^*∗*^
I	0.458 ^*∗*^ ^*∗*^	0.517 ^*∗*^ ^*∗*^	0.498 ^*∗*^ ^*∗*^	0.422 ^*∗*^ ^*∗*^	0.672 ^*∗*^ ^*∗*^	1	0.652 ^*∗*^ ^*∗*^	0.488 ^*∗*^ ^*∗*^
IA	0.438 ^*∗*^ ^*∗*^	0.466 ^*∗*^ ^*∗*^	0.447 ^*∗*^ ^*∗*^	0.384 ^*∗*^ ^*∗*^	0.609 ^*∗*^ ^*∗*^	0.673 ^*∗*^ ^*∗*^	1	0.738 ^*∗*^ ^*∗*^
IE	0.452 ^*∗*^ ^*∗*^	0.393 ^*∗*^ ^*∗*^	0.386 ^*∗*^ ^*∗*^	0.303 ^*∗*^ ^*∗*^	0.388 ^*∗*^ ^*∗*^	0.488 ^*∗*^ ^*∗*^	0.755 ^*∗*^ ^*∗*^	1

^*∗∗*^indicates the significant correlation at 0.01 level.

**Table 5:** The mediating effect test of innovative PsyCap on personal development and innovative action.

Effect type	Coefficient	Se	t	*p*	LLCI	ULCI
CS⟶CI	0.4398	0.0591	9.8463	0.0000	0.3633	0.5322
CI⟶CIA	0.6892	0.0477	13.3970	0.0000	0.6055	0.7966
CS⟶CIA (control intermediary)	0.1862	0.0512	3.8772	0.0001	0.0877	0.2972
CS⟶CI⟶CIA	Coefficient	BootSe			BootLLCI	BootULCI
	0.3174	0.0398			0.2451	0.4022

**Table 6:** The mediating effect of innovative PsyCap on personal development and innovation effect.

Effect type	Coefficient	Se	*t*	*p*	LLCI	ULCI
CS⟶CI	0.4352	0.0556	9.9801	0.0000	0.2894	0.4877
CI⟶CIE	0.5388	0.0653	7.2790	0.0000	0.4377	0.6088
CS⟶CIE (control intermediary)	0.4122	0.0788	5.5124	0.0000	0.1877	0.4987
CS⟶CI⟶CIE	Coefficient	BootSe			BootLLCI	BootULCI
	0.1988	0.0277			0.2033	0.2983

**Table 7:** The mediating effect of PsyCap on power appointment and innovation action.

Effect type	Coefficient	Se	*t*	*p*	LLCI	ULCI
CP⟶CT	0.4289	0.0377	10.2399	0.0000	0.2799	0.4863
CP⟶CI	0.4552	0.0423	11.5877	0.0000	0.4244	0.6122
CT⟶CIA	0.2877	0.0577	5.1124	0.0000	0.1921	0.3755
CI⟶CIA	0.4988	0.0562	8.2375	0.0000	0.4266	0.5899
CP⟶CIA (control intermediary)	0.0952	0.0564	2.4701	0.0108	0.0346	0.1762
	Coefficient	BootSe			BootLLCI	BootULCI
CP⟶CT⟶CIA	0.1766	0.0112			0.0833	0.1799
CP⟶CI⟶CIA	0.1977	0.0378			0.2153	0.2782

**Table 8:** The mediating effect of PsyCap on power appointment and innovation effect.

Effect type	Coefficient	Se	*t*	*p*	LLCI	ULCI
CP⟶CT	0.4266	0.0373	11.7122	0.0000	0.2755	0.4733
CP⟶CI	0.4722	0.0451	11.5882	0.0000	0.4127	0.4942
CT⟶CIE	0.0988	0.0922	1.3221	0.0974	−0.0502	0.2782
CI⟶CIE	0.5092	0.1033	5.4509	0.0000	0.2893	0.7233
CP⟶CIE	0.2164	0.0722	2.6122	0.0007	0.0481	0.2681
(Control intermediary)	Coefficient	BootSe			BootLLCI	BootULCI
CP⟶CT⟶CIE	0.0461	0.0377			-0.0318	0.0862
CP⟶CI⟶CIE	0.1733	0.0511			0.1882	0.2671

**Table 9:** The mediating effect of PsyCap on participation in decision-making and innovation action.

Effect type	Coefficient	Se	*t*	*p*	LLCI	ULCI
CD⟶CT	0.3754	0.0366	10.6988	0.0000	0.3671	0.4892
CD⟶CI	0.4691	0.0312	11.1544	0.0000	0.3512	0.4844
CT⟶CIA	0.2836	0.0698	6.0926	0.0000	0.2451	0.3745
CI⟶CIA	0.4861	0.0633	8.4509	0.0000	0.3893	0.5733
CD⟶CIA	0.0887	0.0412	2.0154	0.0367	0.0122	0.2361
(Control intermediary)	Coefficient	BootSe			BootLLCI	BootULCI
CD⟶CT⟶CIA	0.0964	0.0347			0.0832	0.1704
CD⟶CI⟶CIA	0.2682	0.0402			0.1709	0.2733

**Table 10:** The mediating effect of PsyCap on participation in decision-making and innovation effect.

Effect type	Coefficient	Se	*t*	*p*	LLCI	ULCI
CD⟶CT	0.4213	0.0365	10.6482	0.0000	0.2633	0.4472
CD⟶CI	0.4188	0.0368	11.1543	0.0000	0.4274	0.4799
CT⟶CIE	0.1093	0.0933	1.2699	0.2102	-0.0722	0.3261
CI⟶CIE	0.5277	0.0877	5.4762	0.0000	0.2694	0.7033
CD⟶CIE	0.1856	0.0721	3.0312	0.0122	0.0643	0.2752
(Control intermediary)	Coefficient	BootSe			BootLLCI	BootULCI
CD⟶CT⟶CIE	0.0487	0.0398			-0.0304	0.0944
CD⟶CI⟶CIE	0.1793	0.0377			0.0866	0.2942

**Table 11:** The mediating effect of task-based PsyCap on work guidance and innovative action.

Effect type	Coefficient	Se	*t*	*p*	LLCI	ULCI
CG⟶CT	0.3892	0.0387	10.9033	0.0000	0.3589	0.4731
CT⟶CIA	0.7122	0.0602	11.3754	0.0000	0.5833	0.8245
CG⟶CIA	0.0683	0.0488	2.0132	0.0442	0.0032	0.1798
(Control intermediary)	Coefficient	BootSe			BootLLCI	BootULCI
CG⟶CT⟶CIA	0.3032	0.0712			0.1755	0.4032

**Table 12:** The mediating effect of task-based PsyCap on work guidance and innovation effect.

Effect type	Coefficient	Se	*t*	*p*	LLCI	ULCI
CG⟶CT	0.3902	0.0394	10.9122	0.0000	0.4211	0.5122
CT⟶CIE	0.5122	0.0812	5.8762	0.0000	0.2755	0.6290
CG⟶CIE	0.2163	0.0688	2.3022	0.0180	0.0411	0.2988
(Control intermediary)	Coefficient	BootSe			BootLLCI	BootULCI
CG⟶CT⟶CIE	0.2089	0.0387			0.1409	0.3011

## Data Availability

The data used to support the findings of this study are included within the article.
